# Supplementary material for: Resolving the full spectrum of human genome variation using Linked-Reads
Source: Genome Res. 2019 Apr;29(4):635–45. doi: 10.1101/gr.234443.118 (PMC6442396; doi:10.1101/gr.234443.118)
Supplement: Supplemental Material [file supp_gr.234443.118_Supplemental_longranger-2.2.zip › longranger-2.2.tar.bz2!/lr2.2-public/lib/stan/cmdstan/stan/doc/api/html/classstan_1_1mcmc_1_1sample.html]

Stan: stan::mcmc::sample Class Reference


|  |  |
| --- | --- |
|  | Stan  2.5.0  probability, sampling & optimization |


- Main Page
- Related Pages
- Namespaces
- Classes
- Files

- Class List
- Class Index
- Class Hierarchy
- Class Members

All Classes Namespaces Files Functions Variables Typedefs Enumerations Enumerator Friends Macros Pages

- stan
- mcmc
- sample

Public Member Functions |
List of all members

stan::mcmc::sample Class Reference

`#include <sample.hpp>`

|  |  |
| --- | --- |
| Public Member Functions | |
|  | sample (const Eigen::VectorXd &q, double log\_prob, double stat) |
|  | |
| virtual | ~sample () |
|  | |
| int | size\_cont () const |
|  | |
| double | cont\_params (int k) const |
|  | |
| void | cont\_params (Eigen::VectorXd &x) const |
|  | |
| const Eigen::VectorXd & | cont\_params () const |
|  | |
| double | log\_prob () const |
|  | |
| double | accept\_stat () const |
|  | |
| void | get\_sample\_param\_names (std::vector< std::string > &names) |
|  | |
| void | get\_sample\_params (std::vector< double > &values) |
|  | |

## Detailed Description

Definition at line 13 of file sample.hpp.

## Constructor & Destructor Documentation

|  |  |  |  |
| --- | --- | --- | --- |
| stan::mcmc::sample::sample | ( | const Eigen::VectorXd & | *q*, |
|  |  | double | *log\_prob*, |
|  |  | double | *stat* |
|  | ) |  |  |

inline

Definition at line 17 of file sample.hpp.

|  |  |  |  |  |
| --- | --- | --- | --- | --- |
| virtual stan::mcmc::sample::~sample | ( |  | ) |  |

inlinevirtual

Definition at line 24 of file sample.hpp.

## Member Function Documentation

|  |  |  |  |  |
| --- | --- | --- | --- | --- |
| double stan::mcmc::sample::accept\_stat | ( |  | ) | const |

inline

Definition at line 46 of file sample.hpp.

|  |  |  |  |  |  |
| --- | --- | --- | --- | --- | --- |
| double stan::mcmc::sample::cont\_params | ( | int | *k* | ) | const |

inline

Definition at line 30 of file sample.hpp.

|  |  |  |  |  |  |
| --- | --- | --- | --- | --- | --- |
| void stan::mcmc::sample::cont\_params | ( | Eigen::VectorXd & | *x* | ) | const |

inline

Definition at line 34 of file sample.hpp.

|  |  |  |  |  |
| --- | --- | --- | --- | --- |
| const Eigen::VectorXd& stan::mcmc::sample::cont\_params | ( |  | ) | const |

inline

Definition at line 38 of file sample.hpp.

|  |  |  |  |  |  |
| --- | --- | --- | --- | --- | --- |
| void stan::mcmc::sample::get\_sample\_param\_names | ( | std::vector< std::string > & | *names* | ) |  |

inline

Definition at line 50 of file sample.hpp.

|  |  |  |  |  |  |
| --- | --- | --- | --- | --- | --- |
| void stan::mcmc::sample::get\_sample\_params | ( | std::vector< double > & | *values* | ) |  |

inline

Definition at line 55 of file sample.hpp.

|  |  |  |  |  |
| --- | --- | --- | --- | --- |
| double stan::mcmc::sample::log\_prob | ( |  | ) | const |

inline

Definition at line 42 of file sample.hpp.

|  |  |  |  |  |
| --- | --- | --- | --- | --- |
| int stan::mcmc::sample::size\_cont | ( |  | ) | const |

inline

Definition at line 26 of file sample.hpp.

---

The documentation for this class was generated from the following file:

- src/stan/mcmc/sample.hpp


---

[ Stan Home Page ]

*© 2011–2014,
Stan Development Team.*
